# Inequalities in Open Source Software Development: Analysis of Contributor’s Commits in Apache Software Foundation Projects

**DOI:** 10.1371/journal.pone.0152976

**Published:** 2016-04-20

**Authors:** Tadeusz Chełkowski, Peter Gloor, Dariusz Jemielniak

**Affiliations:** 1 Kozminski University, Warsaw, Poland; 2 Massachusetts Institute of Technology, Center for Cognitive Intelligence, Cambridge, Massachusetts, United States of America; 3 Kozminski University, New Research on Digital Societies (NeRDS) group, Warsaw, Poland; CNRS UMR7622 & University Paris 6 Pierre-et-Marie-Curie, FRANCE

## Abstract

While researchers are becoming increasingly interested in studying OSS phenomenon, there is still a small number of studies analyzing larger samples of projects investigating the structure of activities among OSS developers. The significant amount of information that has been gathered in the publicly available open-source software repositories and mailing-list archives offers an opportunity to analyze projects structures and participant involvement. In this article, using on commits data from 263 Apache projects repositories (nearly all), we show that although OSS development is often described as collaborative, but it in fact predominantly relies on radically solitary input and individual, non-collaborative contributions. We also show, in the first published study of this magnitude, that the engagement of contributors is based on a power-law distribution.

## Introduction

Open collaboration communities have been in the limelight of organization and information studies for the last decade [1]. Open collaboration, in principle, is a way of developing a product collectively, by the use of bottom-up collective intelligence [2] relying on self-organizing communities [3] “open” for anyone to join (or quit), and thus lacking the traditional thresholds of employment and the traditional fears of being fired.

In a famous metaphor introduced by Eric S. Raymond [4], the traditional model can be compared to a medieval cathedral building with top-down management and hierarchy, while the open-collaboration model resembles a bazaar with an a-hierarchical structure without a coordinating center, which still is very successful. Even though not they are not physically present in the same place, software developers involved in Open Source Software (OSS) can create large-scale software [5].

“Open source can be seen as a movement, where communities of highly skilled programmers collectively develop software, often of a quality that outperforms commercial proprietary software” [6]. Indeed, the triumphs of Linux, MySQL, Firefox, and Wordpress speak for themselves. One of the most prominent examples of successful open-software projects is also Apache—absolutely dominating as web server software (running nearly half of all servers worldwide). Open collaboration is sometimes called peer production [7,8,9]. This perspective also emphasizes the equal and a-hierarchical character of open-source development [10,11].

While some authors criticize open-collaboration and peer-production phenomenon as leading to deterioration of quality [12,13], or as resulting in exploitation of participants and creating new inequalities [14,15,16], many others see its great promise [17]. According to Yochai Benkler [18,19], peer production has the potential to redefine capitalism and create a new mode of goods development and consumption with an anti-bureaucratic and a-hierarchical organization of work.

Whether these revolutionary results can in fact be initiated by open collaboration projects remains to be seen [9]. Yet, it is clear that these approaches, at least, rhetorically assume that the phenomenon they are describing relies, in fact, on “collaboration” and “peers”. While some authors are critical of such newspeak [20], it is generally assumed that “collaboration generally happens within the context of a particular production goal; in other words, open collaboration is about people trying to make something together” [1]. As we will show in this article, this presumption is not necessarily valid. From the perspective of code commitment, the processes covered by terms of “open collaboration” or “peer production” are mostly not, in fact, collaborative at all. Instead of a network of peers, they rely on a collection of separate individuals focused on their own goals and ambitions.

Moreover, the participation of contributors is following a steep power law distribution. It is worth noting that open collaboration communities in general follow the “1-9-90 rule” [21,22], under which only 1% of community members actively produce content, 9% are generally somewhat active, and the remaining 90% are passive lurkers. This rule has been widely accepted as valid in open-software projects based on smaller studies. Our findings show that even among the professional and committed contributors, participation is similarly unequal. This finding is significant as we are able to confirm a wide assumption using on an analysis conducted on unprecedented scale (virtually all projects of a major, leading open-source initiative are taken into account). We are able to further ground this finding in an analysis if Gini indexes (counting disparities of commitments) between projects.

## Open-Source Contributors

Open-source contributors can be divided into five groups based on the nature of their involvement. Core developers are responsible for technical concepts and key code commitment. Maintainers are responsible for keeping the project up to date, including porting and compatibility. Patchers actively respond to problems, fixing the product issues. Bug reporters provide issue descriptions. Finally, documenters play the role of power users, supporting others with documentation and instructions [23,24]. Researchers have also examined that, in terms of active participation, North America and Europe are the top regions for Open-Source developers [25]. Self-report studies have measured individual developers’ time commitments, discovering differences in time spent between project leaders (14.13 hours/week), developers (11.10 hours/week) and bug fixers (5.6 hours/week) [26]. In addition to the time spent on development, researchers studied the amount of time community members spent on supporting forums, finding that it may take up 1.5 hours per week [27], and that helping other members is a significant part of software development [28]. On the other hand, Robles and Gonzalez-Barahona have explored the commits distribution in project MONO characterizing commits vs. time and authorship attribution [29], finding high inequalities in the level of commits between different participants. Some researchers have advanced an understanding of the commits distribution on the single-project level (project Ximian Evolution), providing another interesting example of the high inequalities among developers’ commits. “From a total of 196 developers, 5 account for 47% of the MRs, while 20 account for 81% of the MRs, and 55 have done 95% of them” [30], where As defined by German and Mockus, “MR is a logical change of software”. High inequalities have been also confirmed by the GNOME project studies where “[t]he number of checkins performed by a programmer was in the mean 731 with a standard deviation of 1 857 and a maximum of 23 000” [31]–a checkin is an equivalent of a commit.

The Apache Software Foundation has been the subject of a number of academic studies. Researchers have been mostly interested in individual projects such as the Apache HTTP Server [32,33], Apache Lucene [34], or Apache Ant [35]. MacLean, Knutson have provided a Neo4J graph representation of the commit behavior (Apache Software Foundation developers for 2010 and 2011) [36], and in a study of the Apache community, Gala-Pérez, Robles, González-Barahona, and Herraiz [37] analyzed the ratio of mailing list activity to the total number of commits.

Yet, surprisingly, little research has examined commits distribution among the larger group of the Apache Foundation Open Source projects [38], even though studying one of the most successful peer production projects using a large dataset should allow for the most accurate analysis of the studied phenomenon. Our article presents the first analysis of this sort using data from nearly all Apache projects.

## Motivation, Research Questions, and Hypothesis

The goal of this article is to improve our understanding of the OSS participation distribution by analyzing user commits frequency using a large group of the Apache Foundation Open Source projects.

Research Question:

What is the structure of the Apache Software Foundation projects commits distribution?

Hypothesis:

The contributions in the analyzed Apache Software Foundation projects measured in commits are highly unequal, the vast majority of projects are created by a minor but very active part of the open-source community.

## Research Method

In this section, we discuss the methodology used to analyze the collected data. In order to achieve the aims of this study, this work uses the quantification of the individual contributors’ activity on the project level. For the basic picture and the relationship between commits and contributors we use contingency tables. A contingency table is an widely used scientific research standard developed as a unified analytic approach for the multivariate frequency distribution [39]. For the close examination of open source commits distribution, we measure the statistical dispersion using the Gini coefficient. The Gini coefficient is a well-established single measure of inequalities [40] and a popular method supporting studies such as wealth empirical studies. Like most of the inequalities measures, the Gini index might be derived from the Lorenz curve “Gini is a 1 minus twice the area under the Lorenz curve” [41]. For the purpose of the Gini calculation, however, we use the Gini index relationship to covariance proved by Lerman and Yitzhaki [42]:

$$G=2cov\left[y/F\left(y\right)\right]/\bar{y}$$

The advantage of the Gini index is that it’s an easy-to-interpret ratio analysis method. Gini coefficients range between 0 and 1, where 0 represents complete equality and 1 represents complete inequality. It’s worth mentioning that Gini index limitation—since it’s a relative and not absolute measure—might be misleading (e.g. the Gini index will remain the same for the population of developers where 50% of the participants have no activity and the remaining 50% of the population contributes equally, and the population where 75% of the developers contributes in 25% in the overall project activity, and the remaining 25% contributes the remaining 75%) [43].

## Sample Selection

The open-source software “movement” is represented by the network of collaborating programmers. However, there is no single place integrating all existing open-source projects. Open-source projects exist in a wide variety of social, technical, and licensing structures. Cloud-versioning software and repository services like GitHub integrates 26.9M repositories and 10.9M people (see https://github.com/about/press).

For further analysis we’ve selected only projects from the Apache Software Foundation. The Apache Foundation is one of the oldest open-source development organizations. Since 1999, the Apache Foundation has provided technical governance, including collaboration, licensing, and technical policies, for the project committers (a committer is a developer granted access to an Apache Project). For the purpose of collaborative-code development, Apache committers use the subversion revision control system. The Apache Foundation was sampled for the following reasons: firstly, it contains more than 350 projects (see http://apache.org/foundation/), mostly stable and well-established projects with a unified governance model. Secondly, the vast majority of projects are developed over the years, which gives us an opportunity to analyze the structure over time (e.g. the Apache HTTP Server was founded in 1995). Thirdly, the Apache Foundation supported the development of some of the most well-known open-source projects such as Apache HTTP Server and Apache Open Office. Regardless of the Apache Software Foundation’s long history and significant size, the results of this study should not be generalized beyond the Apache Software Foundation community.

What qualifies as an Apache project is, to some extent, open to debate. Even the Apache Foundation lists 262 projects, in some documents 350, or simply “300+ initiatives” elsewhere (on the very same page they also refer to 278 projects). This includes projects in the incubation phase, as well as defunct ones that may cause obvious distortions in the results. Similarly, we have decided against counting the projects that have merged separately or projects that have just one commit, as in our best judgment they should not qualify. Our approach is typical for this kind of research [44].

## Commit

To analyze the contributor activity distribution, we measure the number of commits submitted by the individual contributors. The collective open-software development process consists of commits submitted by the programmers to the unified project repository supported by the source code versioning software. A commit represents a synchronization/exchange of local changes with a remote project repository and is a submission of the individual programmer’s changes. A source-code modification, such as adding, modifying, or removing lines of code, adding or removing files, changes in the documentation files, are typical examples of commits. Because of the open nature of software repositories and their accessibility, commits have been a subject of numerous software development studies [32,45]. Although many researchers tried to classify the value of commits using their size or a number of received comments, we intend to measure only the contributor’s activity, not the value of their work. [45,46,47].

## Data Source

We use data collected by OpenHub.net (formerly Ohloh)—the open-source projects registry. This article is based on the June 2014 snapshot of the OpenHub database, which contains more than 664 thousand open-source projects. In particular, OpenHub provides descriptive information about projects, including name, main programming language, date of creation. Additionally, the registry provides information about the individual contributors and commits. OpenHub retrieves the project data directly from open-source project repositories using connectors to the most popular source versioning systems such as Git, SubVersion, CVS, Mercurial, and Bazaar. OpenHub integrates project information with a user’s feedback, managing the open-source project contributors’ feedback and community. For the purposes of this article, however, we use only raw commit data without information added by the OpenHub community. The Apache Foundation references OpenHub as the historical raw data source.

## Data Collection

In order to collect the Apache Software Foundation project commits data, we developed a Java-based program that crawls the OpenHub database using the REST-based API provided. Our program queries the OpenHub registry using “Apache” as a project identification key word, then iterates over the result table, searching for the unique project ID. Using the project ID, the program executes additional queries and collects project details such as individual contributors’ commits. The initial query returned not only the open-source project originating from the Apache Foundation, but all related projects that extend, use, or integrate Apache projects. Therefore, for the final analysis we have decided to create unified filtering criteria to prepare a clean dataset.

Filtering criteria:

- The project must be listed as an official Apache foundation project at http://projects.apache.org/. Only projects registered and listed are qualified by the Apache Foundation as the “Apache project”.
- The project must not be qualified as “incubating” by the Apache Software Foundation and its homepage must not be listed under the incubator.apache.org domain. The incubation program has been created for the projects wishing to become a part of the Apache Foundation. Typically, it’s a place to verify external organization donation, making sure that it follows the Apache Software Foundation legal standards. A donated project contains existing code with limited and unverified commits information. Thus, projects listed as a part of the incubation are not considered valid date entries for this study. Additionally, the incubation process can lead to project rejection, and a project may not be established as a full Apache member.
- The project must not be qualified by the Apache Software Foundation as discontinued (“moved to attic”). The Apache Software Foundation has created an “attic” project category to manage issues with project life end. It is intended to provide a controlled process to close the project without the active committers or committers that are unable to fulfill their duties. It is common that projects classified as “attic” are merged and integrated with other projects, therefore their commits might be included in other projects.
- Additionally, we have removed 77 records without a proper user name. For selected cases, a detailed review of the removed cases indicated that it belongs to “anonymous”, “none”, “user name”, “unknown”, “root” users, e.g. representing the technical accounts used for the project’s migration process.

**Fig 1:**
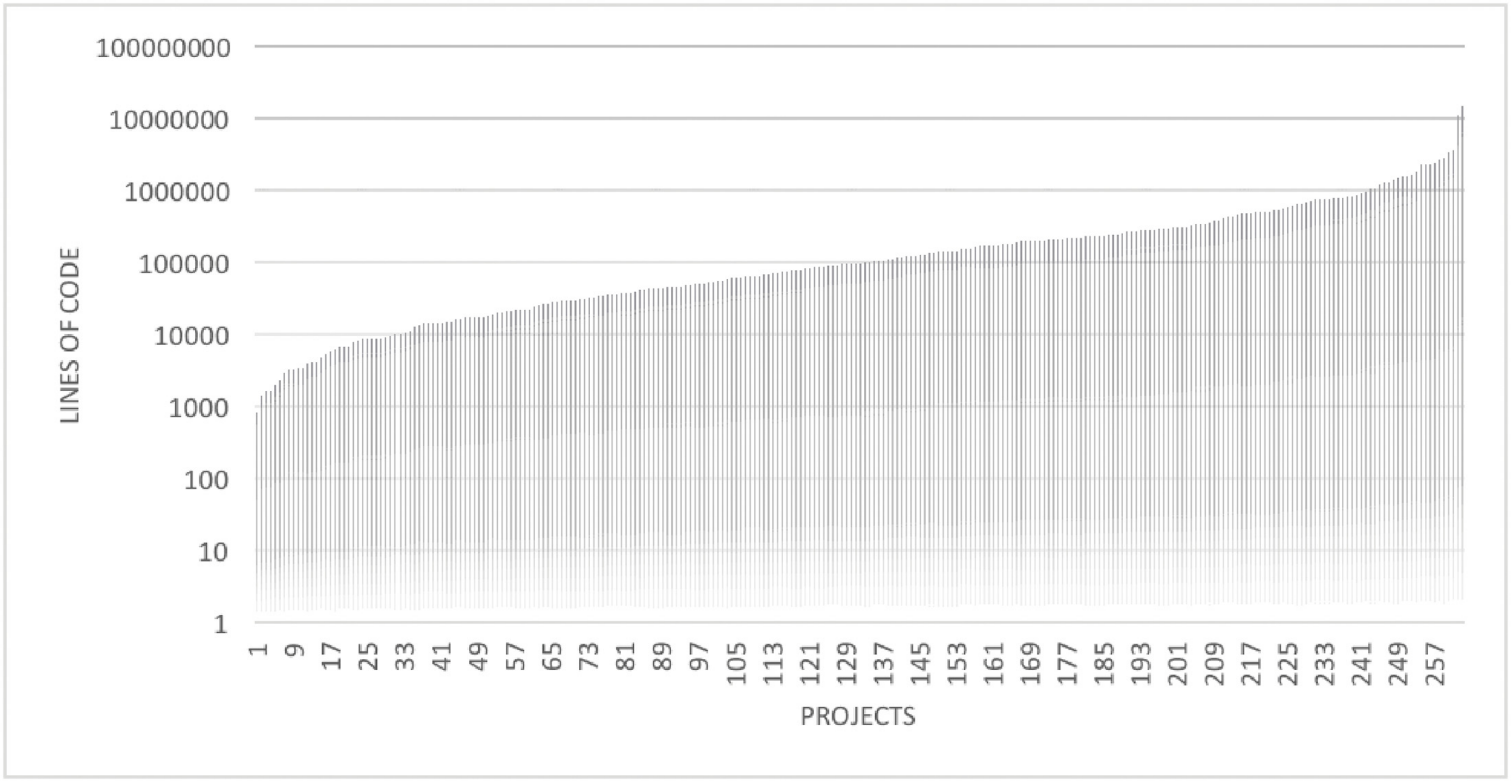
Projects sizes measured as lines of code.

Finally, the collected data encompasses 1,348,405 individual commits. The selected 263 Apache Projects represent 10, 045,099 lines of the source code, which have been created by the 4,661 unique committer accounts (one contributor can commit to multiple projects—see Table 1 and Fig 1).

**Table 1:** Sample commit record retrieved from OpenHub.

Project name	Contributor	Commits
Apache Jackrabbit	Angela Schreiber	1,499

The analyzed projects vary in commits size and contributors amount (Table 2).

**Table 2:** Basic statistics of the analyzed dataset.

Attribute	Total code lines	Total Commits	Contributors
Minimum	822	16	3
Mean	399,411.02	5,127.02	38.30
Median	97,753	1,993	19
1 Quartile	28,459	639	11
3 Quartile	300,162	5,509	35
Maximum	14,625,904	94,585	527
Standard deviation	1,237,915.25	9,710,75	35

## Data and Results Verification

In order to verify the data source (Open Hub), we have selected a set of projects and conducted a manual verification of the OpenHub data with the projects repositories. Data collected automatically has been compared to the commit records inside the projects repository. The only inconsistency we found was that the code collection by OpenHub was delayed compared to the data inside the project repositories.

Additionally, for the project-list validation we reviewed the official Apache project list, making sure that only the Apache projects and its version have been selected for the analysis.

Finally, we matched the individual data records against selected contributors to validate the accuracy of the collected data. We interviewed three developers, and during the interview we presented the commits records and asked for confirmation of the data accuracy. All of the interviewed developers confirmed their commits records.

## Results

**Fig 2:**
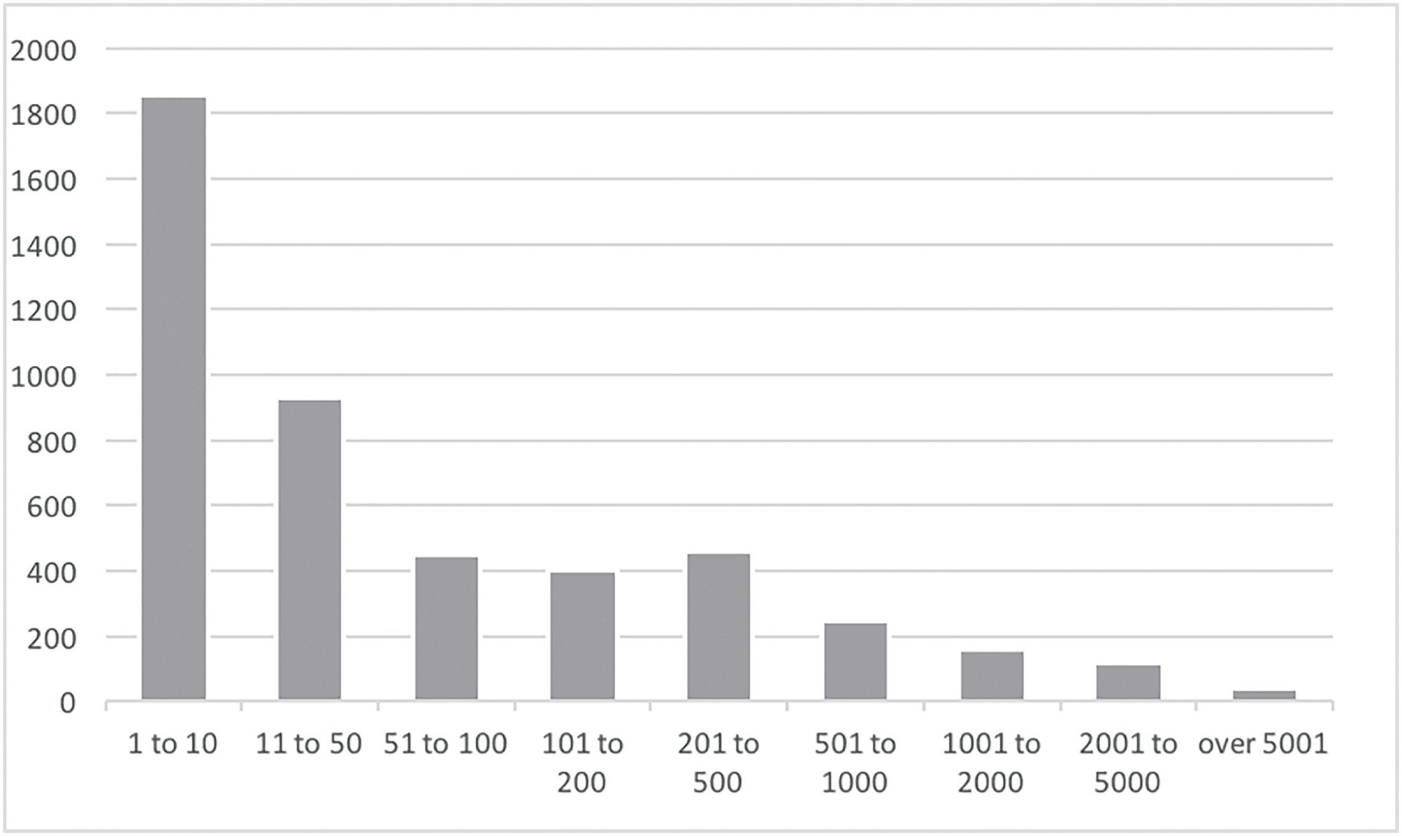
Histogram commits distribution among contributors.

The descriptive analysis (Table 3) of the analyzed projects shows a highly unequal distribution of commits among contributors. Additionally, skewness, a metric of asymmetry, confirms that the mass of the distribution is concentrated on the left with a long right tail (Fig 2).

**Table 3:** Descriptive analysis of commits among contributors.

N	4,661
Minimum	1
Maximum	19,053
Sum	1,348,405
Mean	289.295
Std. Deviation	968.641
Skewness	8.428

To better understand the data distribution and identify similar data groups in an unsupervised way, we have conducted a cluster analysis using k-means clustering and the JENKS algorithm. Both methods provide similar results. As noted in Table 4, in the nine cluster commit frequency distribution list, significant numbers of committers (85.82%) have been aggregated around the lowest cluster center value (56).

**Table 4:** Commits aggregation using k-means clustering classification.

Cluster no.	Cluster center	Committers	%Committers
1	56	4,000	85.82%
2	737	424	9.10%
3	1,921	131	2.81%
4	3,560.8	65	1.39%
5	6,199.8	30	0.64%
6	9,336	3	0.06%
7	12,269	2	0.04%
8	14,230	5	0.11%
9	19,053	1	0.02%

**Fig 3:**
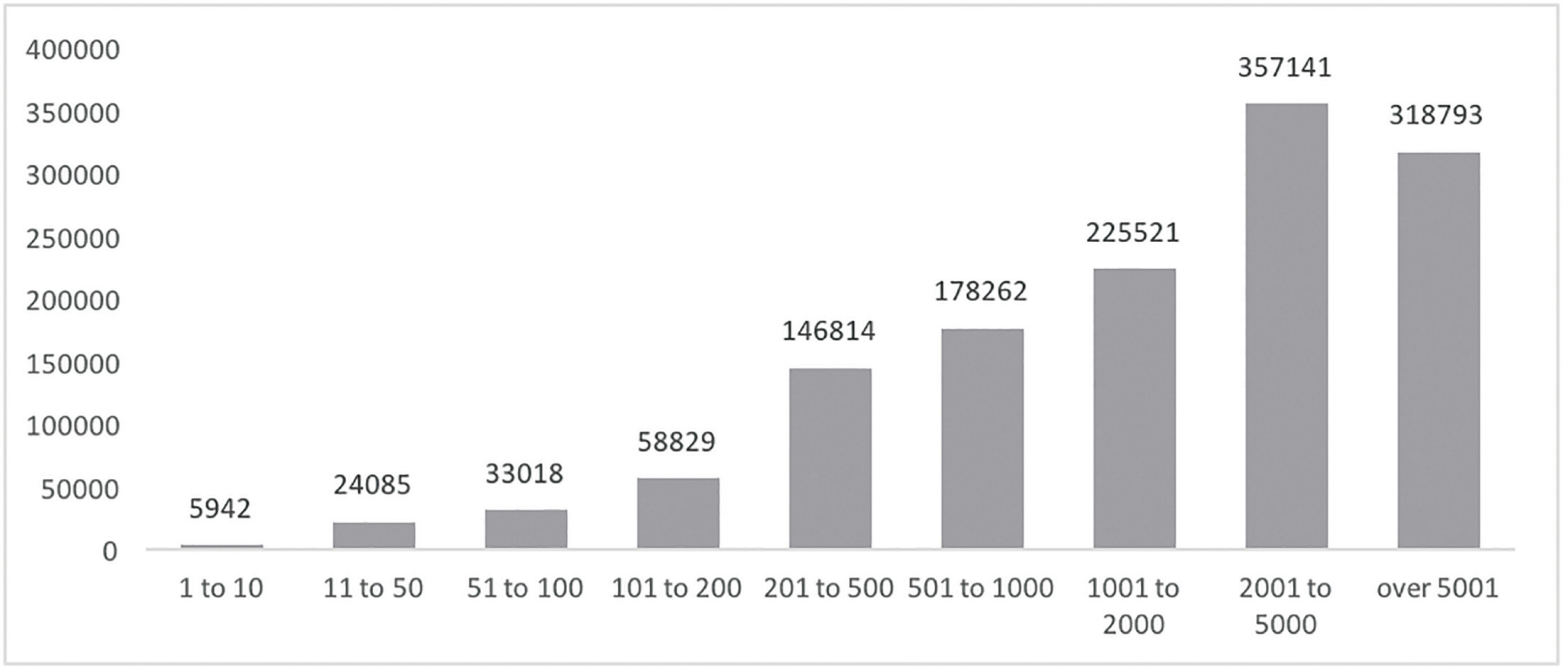
Commits aggregation using expert method.

**Fig 4:**
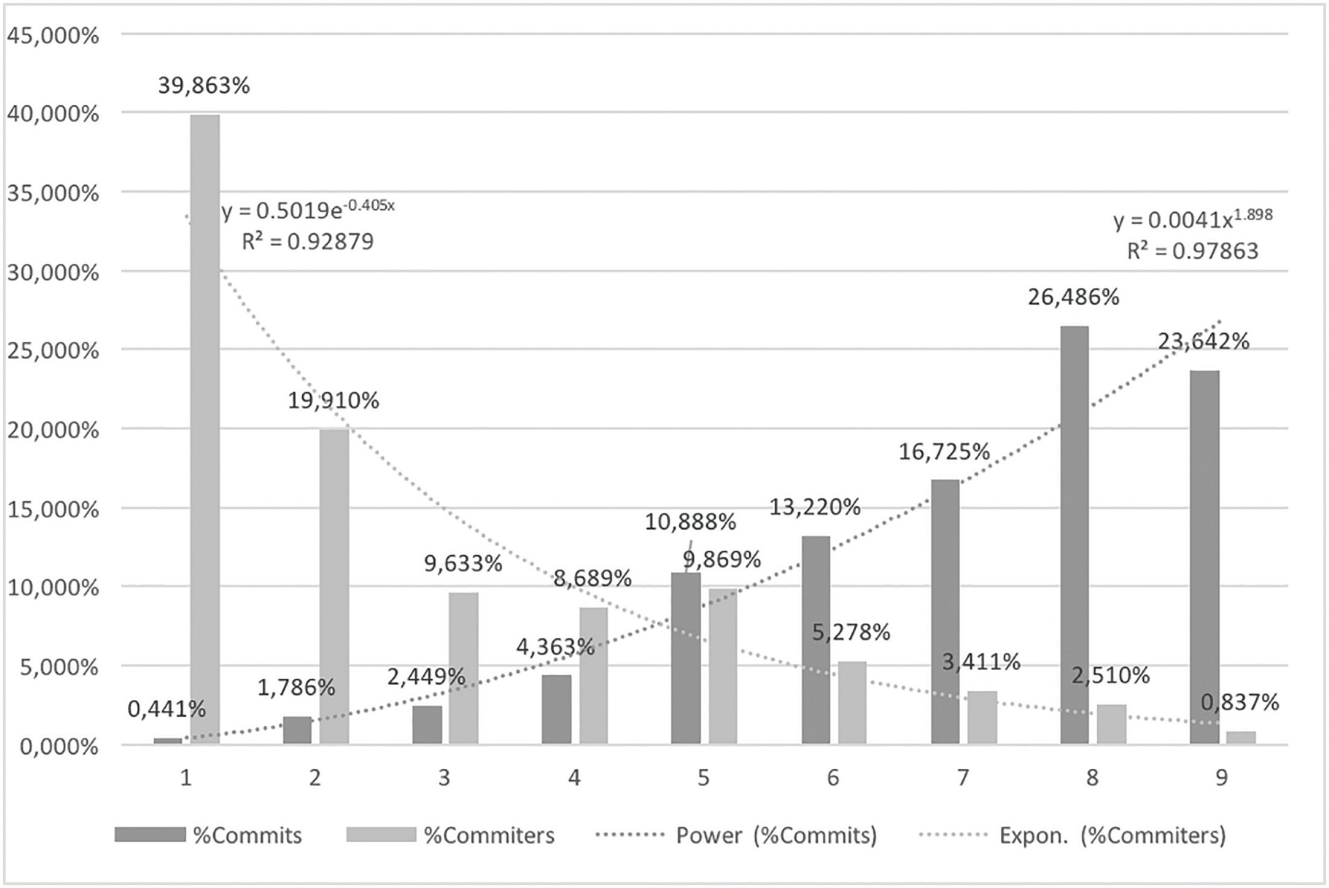
Commits and committers distribution in categories.

For better clarity, we used the expert method (interviews with open-source contributors) to classify nine commit-contribution categories. As presented in Table 5 and Fig 3, 156 committers (the sum of the two top contributing categories), representing only 3.35% of the total analyzed committer’s population, contribute 50.13% of all commits. On the other hand, 2,786 contributors (the sum of the two bottom categories 1–50), representing 59.77% of the population, contribute only 2.27% of the total commits. Fig 4 presents exponential decrease of the committer number for the selected categories and the increase of the commits for the selected categories.

**Table 5:** Commits aggregation using expert method.

Category	Commits	Committers	%Commits	%Committers
1 to 10	5,942	1,858	0.441%	39.863%
11 to 50	24,085	928	1.786%	19.910%
51 to 100	33,018	449	2.449%	9.633%
101 to 200	58,829	405	4.363%	8.689%
201 to 500	146,814	460	10.888%	9.869%
501 to 1000	178,262	246	13.220%	5.278%
1001 to 2000	225,521	159	16.725%	3.411%
2001 to 5000	357,141	117	26.486%	2.510%
over 5001	318,793	39	23.642%	0.837%

## Gini Index Analysis

**Fig 5:**
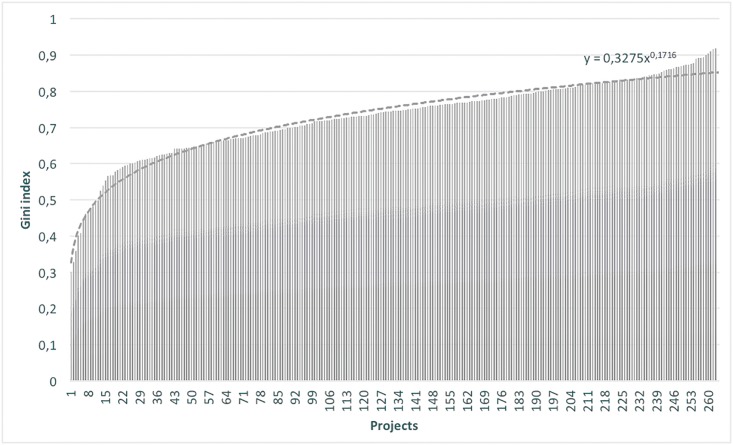
Sorted distribution of Gini index.

**Fig 6:**
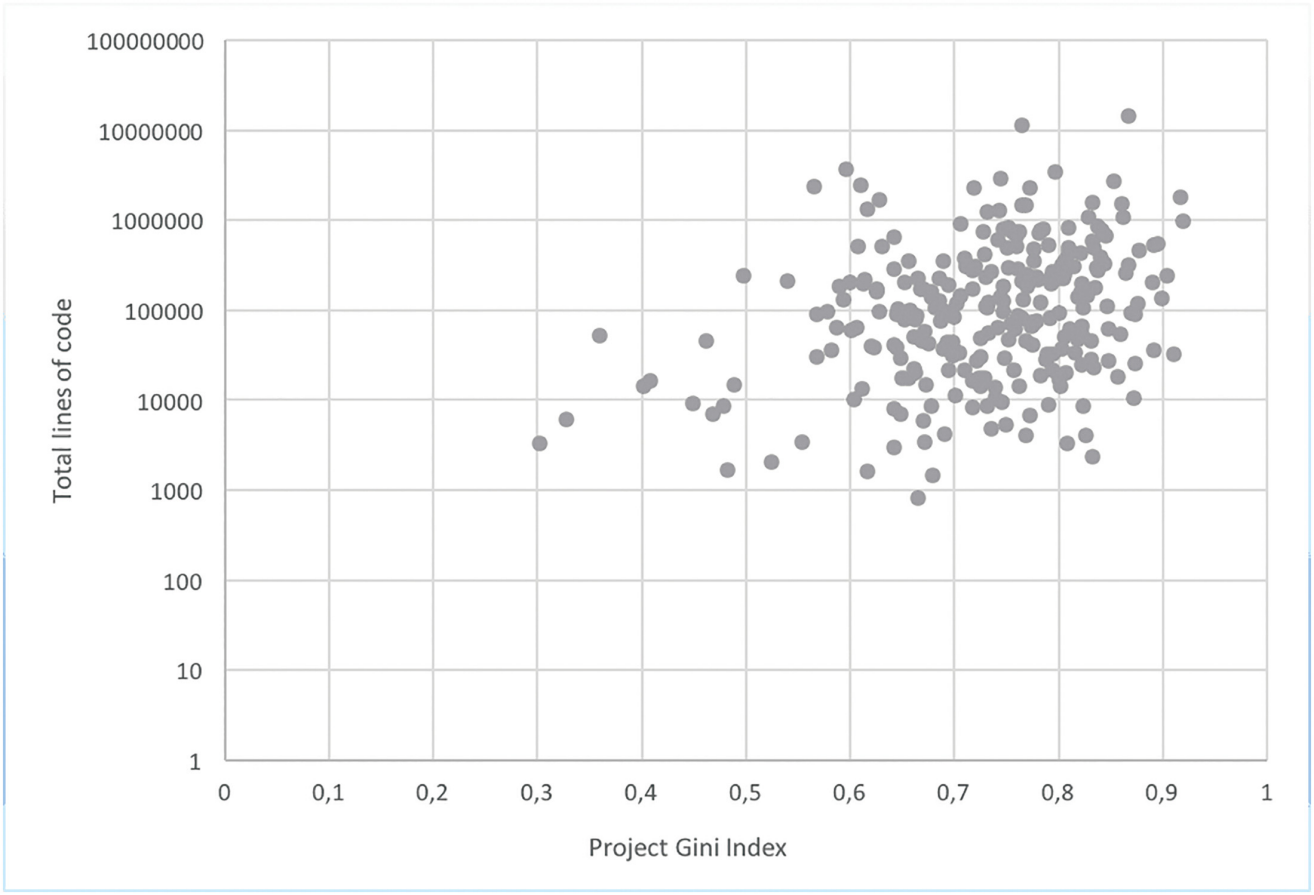
Gini indexes and project size.

**Fig 7:**
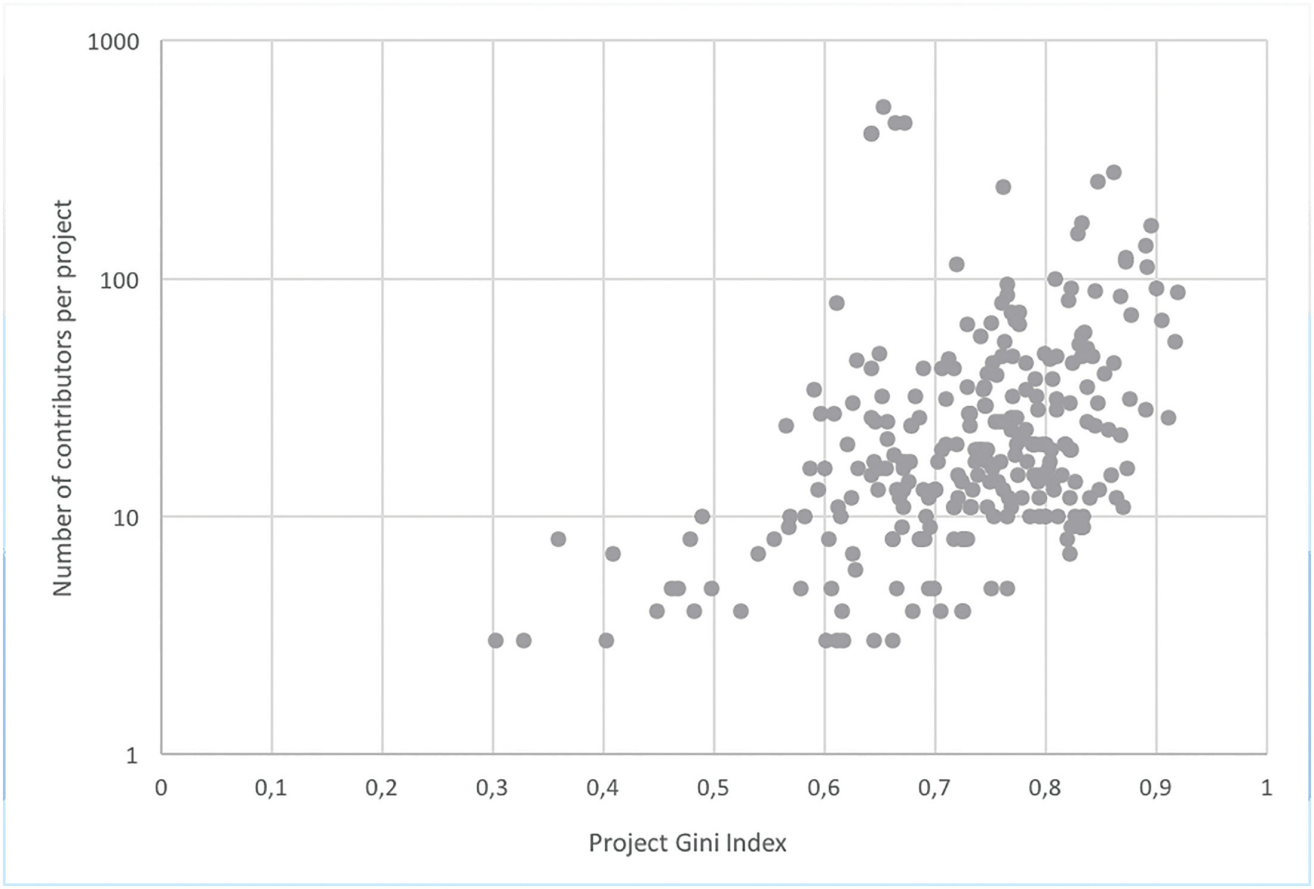
Gini indexes and committers population.

**Fig 8:**
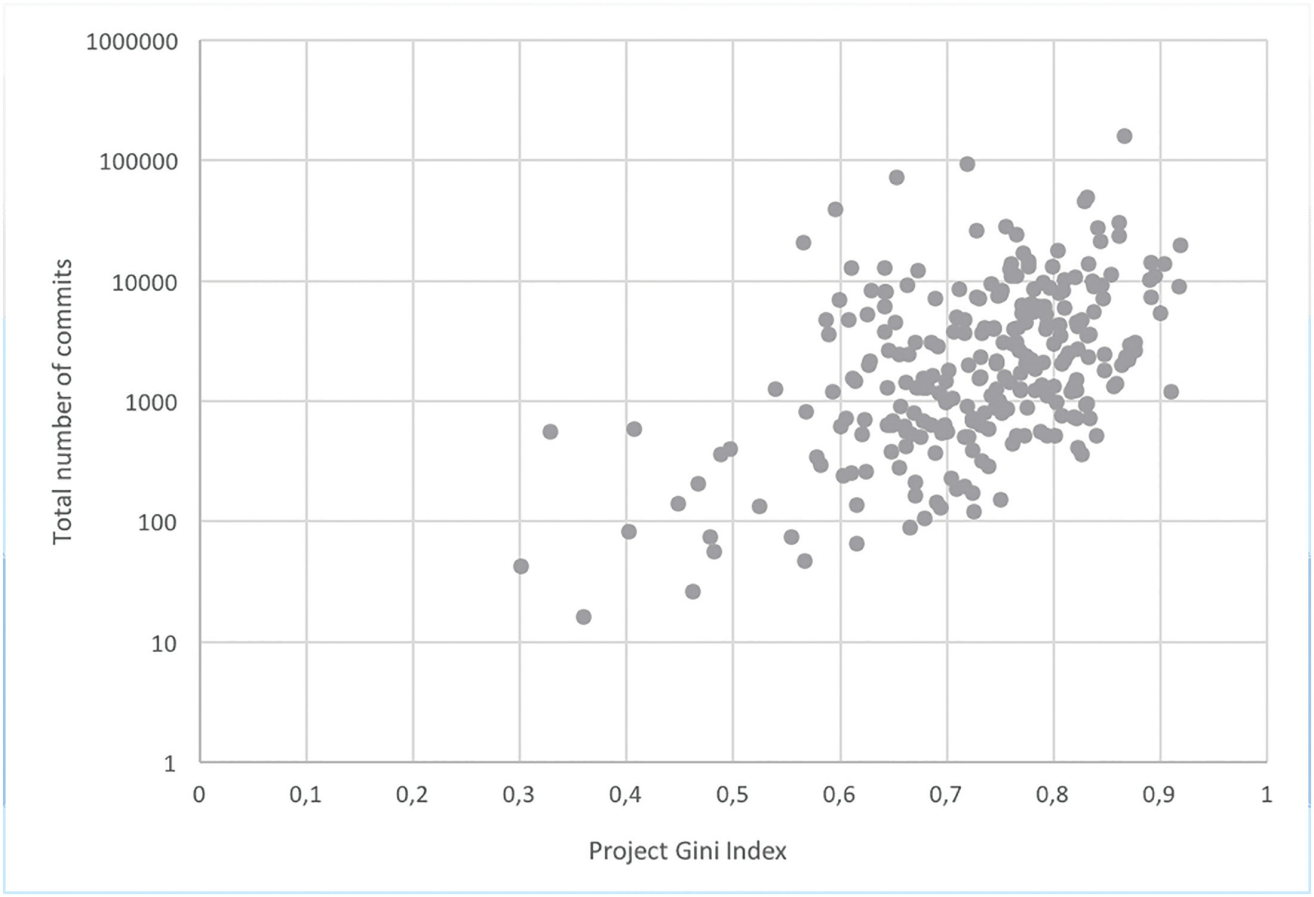
Gini indexes and commits number.

We observe (Fig 5, Tables 6 and 7) high inequalities among the committers’ activities on the project level, measured as Gini index values. Among the 263 analyzed cases, 100 (38.02%) cases are in the range of 0.7–0.8, while 234 (88.97%) of the analyzed population is between 0.6 and 0.9. Additionally, only 9.51% of projects have a Gini value lower than 0.6, and 1.52% are in the range of 0.9 to 1.0. It should be noted that analyzed Gini indexes values are highly concentrated around the mean value. Apache Camel (Gini index 0.919) is the project with the highest level of commit inequality, while Portal JSF Bridge with Gini index = 0.301 has the most equally distributed commits among all of the analyzed projects. Gini-indexes analysis confirms the findings in the contingency-tables analysis. We were unable to find any particular correlation between Gini index value and project size measured as the total lines of code (r = 0.1189), Gini index and project size measured as the number of participating contributors (r = 0.1255), as well as Gini index and project size measured as the number of commits (r = 0.1658). The distribution of Gini indexes and the relationship to project sizes is presented in Figs 6, 7 and 8.

**Table 6:** Properties of the projects Gini Indexes.

Attribute	Gini index of the analyzed 263 projects
Minimum	0.301587302
Mean	0.728541383
Median	0.745753403
1 quartile	0.667293233
3 quartile	0.805603143
Maximum	0.919309711
Standard deviation	0.106680995
Variance	0.013348064

**Table 7:** Distribution of the Gini Indexes.

Gini index value	Number of projects	# of the projects	Accumulated number of the project	Accumulated % of the projects
0.0 <x< = 0.4	3	1.14%	3	1.141%
0.4 <x< = 0.5	9	3.42%	12	4.563%
0.5 <x< = 0.6	13	4.94%	25	9.506%
0.6 <x< = 0.7	66	25.10%	91	34.601%
0.7 <x< = 0.8	100	38.02%	191	72.624%
0.8 <x< = 0.9	68	25.86%	259	98.479%
0.9 <x< = 1.0	4	1.52%	263	100.000%

## Social Network Analysis

**Fig 9:**
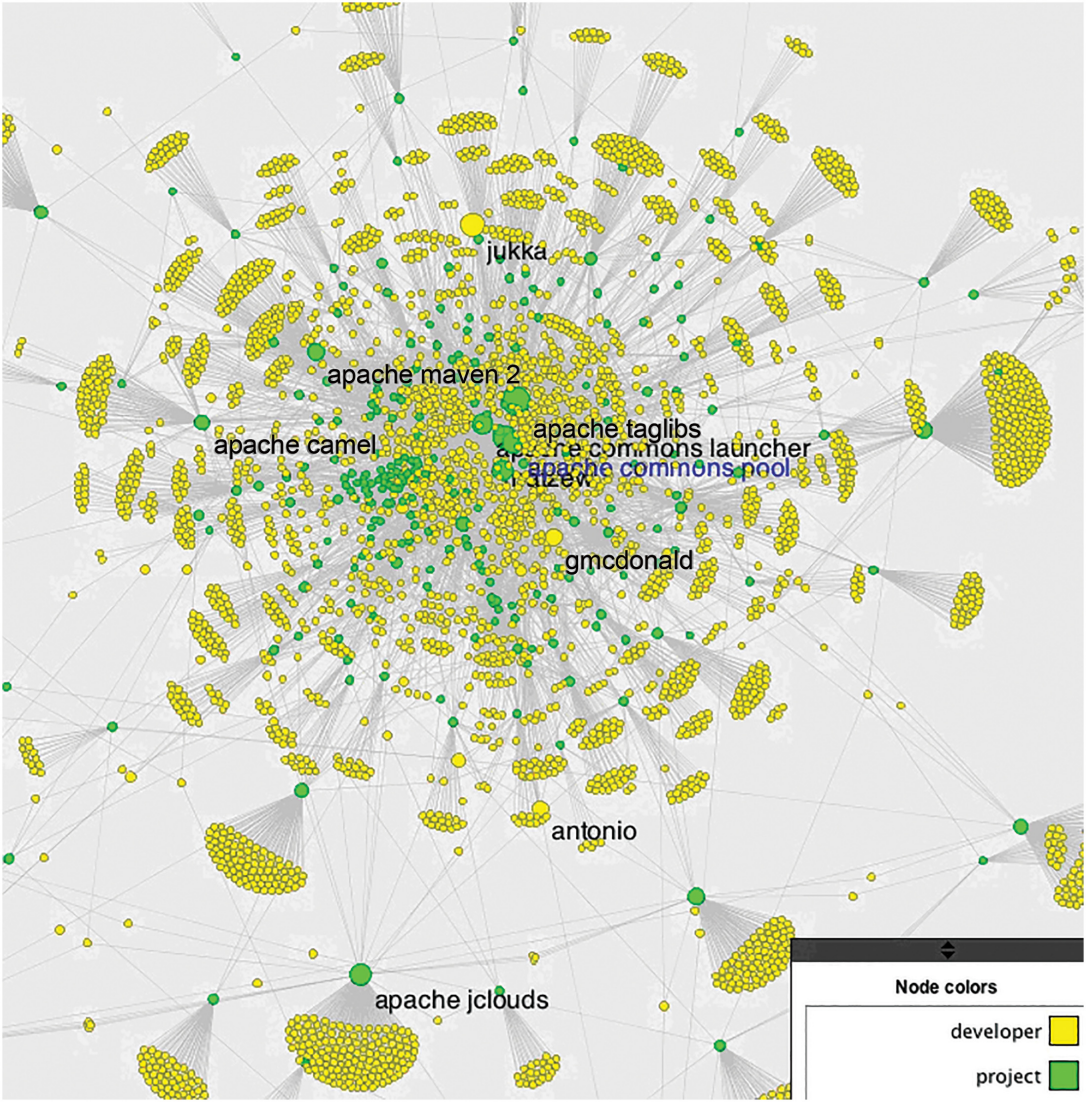
Bipartite graph illustrating the contributor and project network (4924 actors, 4661 developers, 263 projects).

We also conducted a social network analysis of the contributor and project networks by constructing a bipartite graph (Fig 9). The network has been constructed by showing all links between the 4,661 developers and the 263 projects on which they are working. In this bipartite graph we calculate betweenness centrality (Freeman 1977) as a proxy for importance of the developers, as well as a proxy for the importance of the projects. We find that Apache Taglibs has the highest betweenness centrality among the analyzed cases (see Tables 8 and 9). It’s a mature and well-established open-source project, the first code contribution was committed over 15 years ago in September 2000. Over the years, 527 contributors have developed it. Apache Taglibs supports Java Server Pages (JSP). JSP it’s a popular technology simplifying the web application development in Java programming language, and in recent years has became a standard for Java-based web applications. Apache Taglibs is a custom JSP tags library project, which makes it easier for other developers to join the collaborative development effort since their commitments can be easily separated and are more modular than in other projects. We believe that a combination of the three above-mentioned characteristics—mature and well-established project, popular technology, and the modular nature of the Apache Taglibs—are the reasons behind the highest number of contributors, and also indirectly the reason for the highest betweenness centrality among the analyzed projects. When correlating betweenness centrality of projects in the network graph with number of lines, number of committers, and number of commits of the project, we find significant correlation between number of developers and betweenness of a project in the graph (r = 0.907, p<0.001, N = 263). The correlation between commits and project betweenness is r = 0.471 (p<0.001), while the correlation between number of lines and betweenness of the project is r = 0.168 (p = 0.005). This result is not surprising, as we are constructing our network based on the number of people simultaneously working on more than one network, and the more people that work on a project, the more central it becomes. If there is one insight from this short analysis, it is that it is quality of the code matters more than the quantity measured through number of lines or number of commits. It seems that having many eyeballs involved is the best way to increase the influence of a project.

**Table 8:** The top 15 projects by betweenness. We also looked at project size and number of collaborators.

Betweenness centrality	Project name	# of lines	#Commits	#Committers
1,734,235.578	Apache Taglibs	77,397	68,179	527
1,333,166.445	Apache Shale Framework	85,645	9,163	451
1,239,350.732	Apache Cloudstack	1,540,264	23,520	279
1,223,937.202	Apache Spark	109,532	7,055	255
1,156,618.107	Apache Commons Pool	14,702	12,173	447
1,134,737.378	Apache Jclouds	546,572	11,012	166
1,089,733.276	Cordova-Android	25,617	2,552	122
1,075,281.033	Apache Commons Launcher	2,992	7,954	406
1,073,539.122	Apache Commons Modeler	7,981	7,945	405
790,400.008	Apache Maven 2	1,065,693	46,020	155
763,373.662	Apache Libcloud	133,591	5,446	91
761,588.927	Apache Subversion	592,060	49,995	170
626791,9109	Apache Camel	959,655	19,945	87
593164,5311	Apache Gump	36,250	14,181	137
566926,3238	Apache Traffic Server	536,615	7,408	111

**Table 9:** Top 15 committers by betweenness. We also compared their number of commits and number of lines of code they contributed.

Project name	Betweenness centrality	Commits
jukka	1,386,508.632	6,345
joes	919,986.125	1,562
gmcdonald	823,993.580	474
antonio	682,878.895	2,947
joe schaefer	611,604.685	49
gavin mcdonald	527,732,533	39
bdelacretaz	470,439.078	2,476
carlos	372815.024	1,461
niq	358,758.946	1,735
jim	317,239.08	4,972
ashutosh	269,789.440	13
bayard	252,713.137	2,720
sebb	226,232.766	14,447
jesse	223,974.211	1,091
tomwhite	221,081.369	642

As for the Social Network Analysis of the developer, we found that user “jukka”, with 6,345 commits, is the developer with the highest betweenness centrality. Real user "jukka” is a combined record of the accounts “jukka” with 3,208 commits, “Jukka Zitting” with 3,133 commits, and “Jukka Lauri Zitting” with 4 commits, which we have identified as accounts all represented by the same person. A close examination of the project commit logs revealed that “jukka” contributed to 20 projects, including Apache Jackrabbit, Apache Sling, Apache Taglibs, and a number of Apache Commons projects that developers commonly use as a foundational component of other projects. The correlation between the number of commits of a developer and their betweenness centrality is r = 0.222 (p<0.001, N = 4660), which means there is a significant—but not strong—correlation. For instance, user sebb, with 14,447 commits, was well above jukka but has a much lower betweenness. Taking the number of commits as a metric of activity of a developer, we find that the most active developers are not necessarily the most central ones. Rather, we find that there are developers in the core of the social network who, with comparatively few commits, are highly central.

## Discussion

Our study findings undermine the widespread idealistic belief that open-source development is a wide collaborative movement. Rather, we show that in the analyzed Apache Software foundation projects were created by a small, but very active, group of individual, separate contributors.

We conclude that the analyzed Apache Foundation projects experience high levels of inequalities in contributors’ activities measured as commits. The contingency table analysis shows that a small group of contributors is responsible for the majority of commits, which is reinforced by the high levels of the Gini indices among the analyzed projects regardless of project size and committer population.

One main advantage of our research is the analyzed group of projects. The selected 263 cases represent a homogenous group of Apache Software Foundation projects developed under the highly respected Apache Foundation brand. Apache Foundation projects are considered to be among the best organized and the most reliable projects among all OSS projects.

One of the potential issues of our methodology is the semantic association of the commit with the individual programmer’s project contribution. Although commits have been widely used in similar analysis and represent a fundamental element of open-source development, commits are not the only type of open-source collaboration. Community members contribute to open-source development by a number of supporting activities such as reading and answering users’ support questions, preparing technical documentation, or speaking at conferences. Additionally it could be argued that commits might not represent the actual project contribution of a developer. However, the other well-known alternative method of measuring the project contribution by calculating lines of codes has serious flaws and gives no information about the value of the contribution—adding hundreds of lines into a project’s documentation branch is treated identical to a small but essential modification of a project’s core component [48,49]. Therefore, a more effective way of calculating a programmer’s contribution—not only activity as presented in this paper—is an issue that merits further investigation.

Our findings confirm the hypothesis that activities of contributors measured as commits (committers) are unequal. In the analyzed 263 Apache projects, a small but very active core group of developers submitted the majority of commits. Similar power law distributions have been observed in online communities, for example in relation to users’ popularity [50] and for user-content generation [51].

## Conclusions

Our results are not that surprising in the larger context of open-software development. While in other non-professional contexts [52,53] of open collaboration, the benefits of participation are much less clear in economic terms, in open-source software, while not payment-related, they are quite obvious [10,54]. A developer participates in a gift culture, develops one’s network, gets recognition for one’s skills, and also can often combine work with some commercial endeavor. This combined model is increasing in popularity [55,56]. Thus, reputation may be a major factor driving people to develop open source [57,58,59]. To build such a reputation, one does not necessarily have to prove one’s teamwork or leadership skills.

In fact, being a lone hero may be an optimal strategy for portfolio building. Also, while there are methodologies for cyber-teams allowing people to work collectively [60,61], open-collaboration communities in general, and open-software development in particular, attract people who avoid hierarchy and prefer individual work [62,63,64].

Our findings support this perspective. Additionally, our results help problematize the overly simplistic view of open-software development as a mainly collaborative endeavor, as described in our introduction. Open collaboration may well be the best thing since sliced bread, but calling it “collaboration” is an over-emphasis. Peer production is mainly a solitary endeavor and relies much less on peers than enthusiasts of open collaboration would like it to believe.

## Supporting Information

S1 File Source Data. Apache Software Foundation Open Source projects source data.
(XLSX)

## References

[1] Forte A, Lampe C (2013) Defining, understanding, and supporting open collaboration lessons from the literature. American Behavioral Scientist 57: 535–547.

[2] Riehle D, Ellenberger J, Menahem T, Mikhailovski B, Natchetoi Y, Naveh B, et al (2009) Open collaboration within corporations using software forges. Software, IEEE 26: 52–58.

[3] Ostrom E (2000) Collective action and the evolution of social norms. The Journal of Economic Perspectives 14: 137–158.

[4] Raymond ES (1999) The cathedral and the bazaar. Knowledge, Technology & Policy 12: 23–49.

[5] Ciesielska M, Petersen G (2013) Boundary object as a trust buffer. The study of an open source code repository. Tamara Journal for Critical Organization Inquiry 11: 5–14.

[6] Ljungberg J (2000) Open source movements as a model for organising. European Journal of Information Systems 9: 208–216.

[7] Benkler Y, Nissenbaum H (2006) Commons‐based Peer Production and Virtue*. Journal of Political Philosophy 14: 394–419.

[8] Bauwens M (2009) Class and capital in peer production. Capital & Class 33: 121–141.

[9] Kreiss D, Finn M, Turner F (2011) The limits of peer production: Some reminders from Max Weber for the network society. New Media & Society 13: 243–259.

[10] Baytiyeh H, Pfaffman J (2010) Open source software: A community of altruists. Computers in Human Behavior 26: 1345–1354.

[11] Bergquist M, Ljungberg J (2001) The power of gifts: organizing social relationships in open source communities. Information Systems Journal 11: 305–320.

[12] Lanier J (2006) Digital Maoism. The Hazards of the New Online Collectivism. The Edge org: retrieved on 6 April 2012.

[13] Keen A (2007) The cult of the amateur: how today's internet is killing our culture. New York: Broadway Business.

[14] Ritzer G, Jurgenson N (2010) Production, Consumption, Prosumption The nature of capitalism in the age of the digital ‘prosumer’. Journal of Consumer Culture 10: 13–36.

[15] O'Neil M (2010) Shirky and Sanger, or the costs of crowdsourcing. Journal of Science Communication 9: 1–6.

[16] O'Neil M (2011) The sociology of critique in Wikipedia. Critical Studies in Peer Production 1: 1–11.

[17] Pouwelse JA, Garbacki P, Epema D, Sips H (2008) Pirates and Samaritans: A decade of measurements on peer production and their implications for net neutrality and copyright. Telecommunications Policy 32: 701–712.

[18] Benkler Y (2002) Coase's Penguin, or, Linux and" The Nature of the Firm". Yale Law Journal 112: 369–446.

[19] Benkler Y (2006) The wealth of networks: how social production transforms markets and freedom. New Haven: Yale University Press xii, 515 p. p.

[20] Van Dijck J, Nieborg D (2009) Wikinomics and its discontents: a critical analysis of Web 2.0 business manifestos. New Media & Society 11: 855–874.

[21] Gabbiadini A, Mari S, Volpato C (2013) Virtual users support forum: do community members really want to help you? Cyberpsychology, Behavior, and Social Networking 16: 285–292. 10.1089/cyber.2012.0412 23530547

[22] Roth S, Kaivo-Oja J, Hirschmann T (2013) Smart regions: Two cases of crowdsourcing for regional development. International Journal of Entrepreneurship and Small Business 20: 272–285.

[23] Ducheneaut N (2005) Socialization in an open source software community: A socio-technical analysis. Computer Supported Cooperative Work (CSCW) 14: 323–368.

[24] Moon JY, Sproull L (2002) Essence of distributed work: The case of the Linux kernel. First Monday 5. Available: http://firstmonday.org/ojs/index.php/fm/article/viewArticle/801/710.

[25] Gonzalez-Barahona JM, Robles G, Andradas-Izquierdo R, Ghosh RA (2008) Geographic origin of libre software developers. Information Economics and Policy 20: 356–363.

[26] Luthiger B (2005) Fun and software development. Proceedings of the First International Conference on Open Source Systems 273–278.

[27] Lakhani KR, Von Hippel E (2003) How open source software works. Research policy 32: 923–943.

[28] Jemielniak D (2009) Time as symbolic currency in knowledge work. Information and Organization 19: 277–293.

[29] Matellán Olivera V (2003) Studying the evolution of libre software projects using publicly available data. 3rd Workshop on Open Source Software Engineering. Available: http://buleria.unileon.es/xmlui/handle/10612/1796: 111–115.

[30] German D, Mockus A (2003) Automating the measurement of open source projects. Proceedings of the 3rd workshop on open source software engineering 63–67.

[31] Koch S, Schneider G (2000) Results from software engineering research into open source development projects using public data. Diskussionspapiere zum Tätigkeitsfeld Informationsverarbeitung und Informationswirtschaft.

[32] Fielding RT (1999) Shared leadership in the Apache project. Communications of the ACM 42: 42–43.

[33] Mockus A, Fielding RT, Herbsleb JD (2002) Two case studies of open source software development: Apache and Mozilla. ACM Transactions on Software Engineering and Methodology (TOSEM) 11: 309–346.

[34] McCandless M, Hatcher E, Gospodnetic O (2010) Lucene in Action: Covers Apache Lucene 3.0. Greenwich, CT: Manning Publications Co.

[35] Oliva GA, Santana FW, de Oliveira KC, de Souza CR, Gerosa MA (2012) Characterizing key developers: a case study with apache ant Collaboration and Technology: Springer pp. 97–112.

[36] MacLean AC, Knutson CD (2013) Apache Commit History in Neo4J Representation.

[37] Gala-Pérez S, Robles G, González-Barahona JM, Herraiz I (2013) Intensive metrics for the study of the evolution of open source projects: Case studies from Apache Software Foundation projects. MSR '13 Proceedings of the 10th Working Conference on Mining Software Repositories 159–168.

[38] Crowston K, Wei K, Howison J, Wiggins A (2012) Free/Libre open-source software development: What we know and what we do not know. ACM Computing Surveys (CSUR) 44: 1–35.

[39] Bishop YM, Fienberg SE, Holland PW (1975) Discrete multivariate analysis: theory and practice. Cambridge, MA: MIT Press.

[40] Cheong K (2000) A new interpretation and derivation of the Gini coefficient. Seoul Journal of Economics 13: 391–406.

[41] Shalit H (1985) PRACTITIONERS'CORNER* Calculating the Gini Index of inequality for Individual Data. Oxford Bulletin of Economics and Statistics 47: 185–189.

[42] Lerman RI, Yitzhaki S (1989) Improving the accuracy of estimates of Gini coefficients. Journal of econometrics 42: 43–47.

[43] De Maio FG (2007) Income inequality measures. Journal of epidemiology and community health 61: 849–852. 17873219 10.1136/jech.2006.052969 PMC2652960

[44] Howison J, Crowston K (2004) The perils and pitfalls of mining SourceForge. Proceedings of the International Workshop on Mining Software Repositories (MSR 2004). Available: http://floss.syr.edu/sites/crowston.syr.edu/files/The%20perils%20and%20pitfalls%20of%20mining%20SourceForge.pdf: 7–11.

[45] Arafat O, Riehle D (2009) The commit size distribution of open source software. HICSS'09 42nd Hawaii International Conference on System Sciences 1–8.

[46] Hebb DO (1946) On the nature of fear. Psychological Review 53: 259 20285975 10.1037/h0061690

[47] Hahsler M, Koch S (2005) Discussion of a large-scale open source data collection methodology. HICSS'05 Proceedings of the 38th Annual Hawaii International Conference on System Sciences

[48] Krishnamurthy S (2002) Cave or community?: An empirical examination of 100 mature open source projects. First Monday 2.

[49] Bird C, Nagappan N (2012) Who? where? what?: examining distributed development in two large open source projects. Proceedings of the 9th IEEE Working Conference on Mining Software Repositories 237–246.

[50] Johnson SL, Faraj S, Kudaravalli S (2014) Emergence of Power Laws in Online Communities: The Role of Social Mechanisms and Preferential Attachment. MIS Quarterly 38: 795–808.

[51] Hargittai E, Walejko G (2008) The Participation Divide: Content creation and sharing in the digital age. Information, Community and Society 11: 239–256.

[52] Jemielniak D (2014) Common knowledge? An ethnography of Wikipedia. Stanford, CA: Stanford University Press.

[53] Hill BM, Monroy-Hernández A (2013) The Remixing Dilemma The Trade-Off Between Generativity and Originality. American Behavioral Scientist 57: 643–663.

[54] Bitzer J, Schrettl W, Schröder PJ (2007) Intrinsic motivation in open source software development. Journal of Comparative Economics 35: 160–169.

[55] Fitzgerald B (2006) The transformation of open source software. MIS Quarterly 30: 587–598.

[56] Riehle D (2012) The single-vendor commercial open course business model. Information Systems and e-Business Management 10: 5–17.

[57] Wasko MM, Faraj S (2005) Why should I share? Examining social capital and knowledge contribution in electronic networks of practice. MIS Quarterly 29: 35–57.

[58] Von Hippel E, Von Krogh G (2003) Open source software and the" private-collective" innovation model: Issues for organization science. Organization Science: 209–223.

[59] Weber S (2004) The Success of Open Source. Cambridge: Harvard University Press.

[60] Gloor PA (2005) Swarm creativity: Competitive advantage through collaborative innovation networks. Oxford: Oxford University Press.

[61] Gloor PA, Cooper SM (2007) The new principles of a swarm business. MIT Sloan Management Review 48: 81–84.

[62] Jemielniak D (2015) Naturally emerging regulation and the danger of delegitimizing conventional leadership: Drawing on the example of Wikipedia In: Bradbury H, editor. The SAGE Handbook of Action Research. London, UK—New Delphi, India—Thousand Oaks, CA: Sage.

[63] Crowston K, Howison J (2006) Hierarchy and centralization in free and open source software team communications. Knowledge, Technology & Policy 18: 65–85.

[64] Demil B, Lecocq X (2006) Neither market nor hierarchy nor network: The emergence of bazaar governance. Organization Studies 27: 1447–1466.

